# Assessment of Ultrasound-Controlled Diagnostic Methods for Thyroid Lesions and Their Associated Costs in a Tertiary University Hospital in Spain

**DOI:** 10.3390/jcm14155551

**Published:** 2025-08-06

**Authors:** Lelia Ruiz-Hernández, Carmen Rosa Hernández-Socorro, Pedro Saavedra, María de la Vega-Pérez, Sergio Ruiz-Santana

**Affiliations:** 1 Biomedicine Research Program, Doctoral School, University of Las Palmas de Gran Canaria, E-35001 Las Palmas de Gran Canaria, Spain; lelia.ruiz101@alu.ulpgc.es; 2 Department of Radiology, Hospital Universitario de Gran Canaria Dr. Negrín, E-35010 Las Palmas de Gran Canaria, Spain; 3 Department of Clinical Sciences, University of Las Palmas de Gran Canaria, E-35016 Las Palmas de Gran Canaria, Spain; 4 Department of Mathematics, University of Las Palmas de Gran Canaria, E-35016 Las Palmas de Gran Canaria, Spain; pedro.saavedra@ulpgc.es; 5 Department of Pathology, Hospital Universitario de Gran Canaria Dr. Negrín, Barranco de la Ballena s/n, E-35010 Las Palmas de Gran Canaria, Spain; mjvegper@gobiernodecanarias.org; 6 Department of Medical and Surgical Sciences, University of Las Palmas de Gran Canaria, E-35016 Las Palmas de Gran Canaria, Spain

**Keywords:** thyroid neoplasms, ultrasonography, diagnostic imaging, biopsy, fine-needle, thyroid, nodule, cost-effectiveness analysis, elastography, superb microvascular imaging, TI-RADS, Bethesda System

## Abstract

**Background/Objectives:** Accurate diagnosis of thyroid cancer is critical but challenging due to overlapping ultrasound (US) features of benign and malignant nodules. This study aimed to evaluate the diagnostic performance of non-invasive and minimally invasive US techniques, including B-mode US, shear wave elastography (SWE), color Doppler, superb microvascular imaging (SMI), and TI-RADS, in patients with suspected thyroid lesions and to assess their reliability and cost effectiveness compared with fine needle aspiration (FNA) biopsy. **Methods:** A prospective, single-center study (October 2023–February 2025) enrolled 300 patients with suspected thyroid cancer at a Spanish tertiary hospital. Of these, 296 patients with confirmed diagnoses underwent B-mode US, SWE, Doppler, SMI, and TI-RADS scoring, followed by US-guided FNA and Bethesda System cytopathology. Lasso-penalized logistic regression and a bootstrap analysis (1000 replicates) were used to develop diagnostic models. A utility function was used to balance diagnostic reliability and cost. **Results:** Thyroid cancer was diagnosed in 25 patients (8.3%). Elastography combined with SMI achieved the highest diagnostic performance (Youden index: 0.69; NPV: 97.4%; PPV: 69.1%), outperforming Doppler-only models. Intranodular vascularization was a significant risk factor, while peripheral vascularization was protective. The utility function showed that, when prioritizing cost, elastography plus SMI was cost effective (α < 0.716) compared with FNA. **Conclusions:** Elastography plus SMI offers a reliable, cost-effective diagnostic rule for thyroid cancer. The utility function aids clinicians in balancing reliability and cost. SMI and generalizability need to be validated in higher prevalence settings.

## 1. Introduction

The diagnosis of serious thyroid conditions, such as thyroid cancer and thyroiditis, is a critical focus in modern medicine and clinical practice [1]. Radiological diagnostic methods, including their performance and associated costs, are increasingly vital in thyroid care worldwide. Historically, no single ultrasound (US) feature or combination of features was considered sufficiently sensitive or specific to reliably identify malignancy due to the significant overlap in US characteristics among benign, borderline, and certain malignant thyroid lesions. Consequently, conventional B-mode US alone was deemed inadequate for accurate thyroid cancer risk stratification [2,3]. However, advancements in ultrasound technology, combined with fine needle aspiration biopsy (FNAB) performed at the optimal time [4], have transformed diagnostic approaches. Today, B-mode US, integrated with newer ultrasound techniques, serves as the cornerstone of imaging for evaluating suspicious thyroid lesions, playing a pivotal role in their diagnosis, management, and future treatment [5].

To accurately diagnose and classify suspicious thyroid nodules or enlargements, and to develop predictive rules or scoring systems, clinicians routinely employ both conventional B-mode ultrasonography and advanced ultrasound techniques. These methods enable a comprehensive evaluation of nodule characteristics, which are scored using systems such as the Thyroid Imaging Reporting and Data System (TI-RADS) [6], color Doppler [7], superb microvascular imaging (SMI) [8], and shear-wave elastography (SWE) [9]. The integration of these ultrasound modalities facilitates the assessment of diagnostic feasibility and associated costs. Subsequently, fine-needle aspiration biopsy (FNAB), typically performed under ultrasound guidance, yields conclusive or inconclusive cytologic results [10].

The Thyroid Imaging Reporting and Data System (TI-RADS) was developed to identify high-risk thyroid nodules for fine-needle aspiration (FNA) cytology. Introduced in 2009 [11], TI-RADS uses B-mode ultrasound features to classify focal thyroid lesions into categories based on increasing risk of thyroid cancer. Since then, the system has been refined by various organizations, including the American [6,12], European [13], and Asian thyroid societies [14,15], with its validity widely evaluated. FNA smears are assessed using the Bethesda System for Reporting Thyroid Cytopathology (TBSRTC), which comprises six diagnostic categories, each associated with a stratified risk of malignancy [16]. A significant correlation exists between TI-RADS ultrasound classification and TBSRTC [17]. When both systems indicate low-risk categories, further clinical procedures may be unnecessary. Conversely, higher risk scores may warrant prompt additional interventions due to an elevated likelihood of malignancy [10].

Although TI-RADS and TBSRTC provide valuable diagnostic guidance, they are not always conclusive. To further stratify indeterminate thyroid nodules as malignant, additional tests are often employed, including multigene molecular testing [18], the detection of circulating carcinoma biomarkers, or advanced imaging techniques, sometimes integrated with artificial intelligence methods [19]. Molecular testing has proven effective in reducing unnecessary surgeries and is considered cost effective in managing indeterminate thyroid nodules [20,21,22].

Advanced radiological techniques, including shear wave elastography (SWE), color Doppler, and superb microvascular imaging (SMI) [23,24], provide comprehensive insights into thyroid nodule characteristics, such as size, composition, and vascularity [2,7,25], aiding in determining their true nature. SWE offers a quantitative measure of thyroid tissue stiffness by assessing the local shear elastic modulus [9], with higher kilopascal values indicating stiffer tissue. Although SWE has not been extensively studied in thyroid lesions, it has shown a high diagnostic yield, closely aligning with histopathologic results for thyroid nodules. As a noninvasive ultrasound technique, SWE enhances risk stratification for malignant thyroid lesions [26,27].

Color Doppler ultrasound and superb microvascular imaging (SMI) are essential tools for evaluating thyroid nodules and optimizing patient-centered outcomes. Color Doppler enhances conventional B-mode ultrasonography by detecting and quantifying blood flow [7], improving the safety of ultrasound-guided fine-needle aspiration or Tru-cut biopsies through detailed vascular assessment. SMI v6.0, a specialized software, visualizes low-velocity blood flow without motion artifacts from adjacent structures, surpassing the capabilities of conventional Doppler techniques [8]. It rapidly confirms low blood flow or its absence. SMI operates in two modes: monochrome (mSMI), which enhances sensitivity by isolating vasculature and suppressing background information, and color (cSMI), which simultaneously displays B-mode and color data [28]. To our knowledge, SMI has not been previously studied in thyroid lesion evaluations.

This study aims to evaluate the diagnostic accuracy of various non-invasive or minimally invasive thyroid ultrasound techniques, individually or in combination (scoring), for identifying suspicious thyroid lesions in patients undergoing ultrasound-guided fine needle aspiration (FNA) followed by cytopathology, the gold standard, at a tertiary referral university hospital in Spain. Additionally, the diagnostic reliability and cost-effectiveness of these techniques are assessed. Furthermore, diagnostic reliability as well as the construction and validation of a cost-effectiveness decision function for these techniques are evaluated.

## 2. Materials and Methods

### 2.1. Study Design and Participants

This prospective, single-center, cohort study conducted from October 2023 to February 2025 enrolled patients with thyroid enlargement initially diagnosed by their general practitioner and referred to the Ultrasound Section of the Radiology Department at a tertiary university hospital in Spain for suspected thyroid cancer. To address challenges in developing diagnostic methods for thyroid cancer, only one observation per patient was analyzed. For patients with multiple observations, we selected the first instance with a pathologically confirmed thyroid lesion diagnosis. If no such diagnosis was available, the earliest visit was included. This approach was chosen due to the relatively low prevalence of malignant thyroid tumors in our region.

### 2.2. Clinical and Imaging Data

Nodule characteristics, including echogenicity, shape, margins, internal echoes, vascularity (intranodular, peripheral, or microvascularization), and elastographic parameters, were evaluated. Each patient underwent conventional B-mode ultrasound, followed by elastography, color Doppler, and superb microvascular imaging (SMI) to assess peripheral and intranodular vascularization. SMI was performed using the Aplio™ 500 ultrasound system (Toshiba Medical Systems Corporation, Tochigi, Japan), which visualizes low-velocity blood flow without requiring contrast medium.

Nodule characteristics were scored according to Thyroid Imaging Reporting and Data System (TI-RADS) criteria. A single expert radiologist, blinded to prior diagnoses, evaluated all nodules. Subsequently, ultrasound-guided fine needle aspiration (FNA) biopsies were performed, and specimens were analyzed by pathology specialists and categorized as thyroiditis, benign, carcinoma, or undetermined. Inadequate or indeterminate specimens were either repeated or excluded from the study.

Patients with conclusive FNA cytopathology results were included, whereas those with nondiagnostic pathology reports (Bethesda category I) were excluded. The Bethesda System for Reporting Thyroid Cytopathology categories and their associated malignancy risks are provided below:

I. Nondiagnostic;
II. Benign (<3%);
III. Atypia of Undetermined Significance or Follicular Lesion of Undetermined Significance (10–30%);
IV. Follicular Neoplasm or Suspicious for Follicular Neoplasm (25–40%);
V. Suspicious for Malignancy (50–75%);
VI. Malignant (97–99%) [29].

### 2.3. Statistical Analysis

#### 2.3.1. Univariate Analysis

Categorical variables are reported as frequencies and percentages, while continuous variables are expressed as means and standard deviations (SDs) for normally distributed data or as medians and interquartile ranges (IQRs, 25th–75th percentile) for non-normally distributed data. Percentages were compared using the Chi-square (χ^2^) test or Fisher’s exact test, as appropriate. Means were compared using the t-test, and medians were compared using the Wilcoxon test for independent data.

#### 2.3.2. Multivariate Logistic Regression

Thyroid cancer prediction models were developed using Lasso-penalized logistic regression (Least Absolute Shrinkage and Selection Operator) [30]. Unlike conventional maximum likelihood estimation, Lasso enhances model parsimony and robustness by simultaneously performing variable selection and regularization through an L1-norm penalty. This approach is particularly effective for high-dimensional datasets or when multicollinearity may affect traditional methods, as it retains only the most predictive covariates (e.g., clinically relevant biomarkers or risk factors) while eliminating noise. Confidence intervals (95%) for estimated coefficients were derived using bootstrap analysis.

The following thyroid cancer prediction models were constructed:

Model based solely on Doppler results.

Model based solely on SMI results.

Model including all variables, with selection performed via Lasso.

Model including all variables except for elastography, with selection performed via Lasso.

#### 2.3.3. Receiver Operating Characteristics

For all predictors of thyroid cancer, a receiver operating characteristic (ROC) analysis was carried out. The area under the corresponding ROC curve was estimated using a 95% confidence interval. The discriminant threshold was determined as the value that maximizes Youden’s J statistic [31]. This is defined as follows:

J = Sensitivity + Specificity − 1

A diagnosis rule was acceptable when J > 0.5.

For all thyroid cancer predictors, accuracy, Youden’s index, sensitivity, specificity, and predictive values were estimated along with their 95% confidence intervals.

Statistical significance was set at *p* < 0.05. Data were analyzed using R software version 4.2.1 [32].

#### 2.3.4. Cost–Benefit Utility Functions

We defined a set of cost–benefit utility functions to evaluate diagnostic rules based on Youden’s index (*J*) [31] and the economic cost (*C*) of the marker [33]. Specifically, we considered a collection of diagnostic rules $D_{i}=\left(J_{i},C_{i}\right):i=1,\ldots ,d$, with $J_{i}$ representing the Youden’s index and $C_{i}$ representing the cost associated with the *i*-th rule.

To enable fair comparison across rules, the costs were normalized as follows: ${\tilde{C}}_{i}=C_{i}/\sum_{k}C_{k}$. We then defined the utility function for each rule as follows:

$$U_{i}=\alpha \cdot J_{i}+\left(1-\alpha \right)\cdot \left(1-{\tilde{C}}_{i}\right):0<\alpha <1$$

where α is a weighting parameter that balances the trade-off between diagnostic reliability (captured by $J_{i}$) and cost efficiency (represented $\left(1-{\tilde{C}}_{i}\right)$. Values of α close to 1 prioritize reliability, while values near 0 emphasize cost minimization. In this framework, a diagnostic rule $D_{i}$ was preferred over another rule $D_{j}$ ($D_{i}≻D_{j}$) and only if $U_{i}>U_{j}$. Formally, the optimal decision rule $D_{opt}$ was defined as the rule that maximizes the utility function.

## 3. Results

This study enrolled 300 patients, of whom 25 (8.3%) were diagnosed with thyroid cancer via fine-needle aspiration (FNA) or tru-cut biopsies with cytopathologic confirmation, 271 (90.3%) had a negative diagnosis, and 4 (1.3%) had an indeterminate diagnosis.

Diagnostic rules were developed based on the 296 patients with confirmed diagnoses. Table 1 summarizes the patient characteristics for the entire cohort (*n* = 296) and subgroups defined by the presence (*n* = 25) or absence (*n* = 271) of thyroid cancer. No statistically significant differences were observed in age or thyroiditis diagnosis between subgroups. However, the cancer group had a significantly higher proportion of men compared with the non-cancer group (44% vs. 23.2%; *p* = 0.022). All other comparisons between subgroups were also statistically significant, as shown in Table 1.

Table 2 presents the coefficients of the logistic regression models, including only Lasso-selected variables. Bootstrap analysis with 1000 replicates was used to calculate 95% confidence intervals and *p*-values. Both Doppler-only and SMI-only models identified intranodular vascularization as a risk factor for thyroid cancer, while peripheral vascularization showed a protective effect in the Doppler peripheral and SMI models (encompassing SMI-detected intranodular and peripheral vascularization). When all variables—TI-RADS, Doppler, SMI, and elastography—were included in the logistic model, Lasso retained elastography (benign/malignant classification) and SMI-detected intranodular vascularization. When elastography was excluded, the final model incorporated SMI-detected intranodular vascularization and TI-RADS.

**Figure 1:**
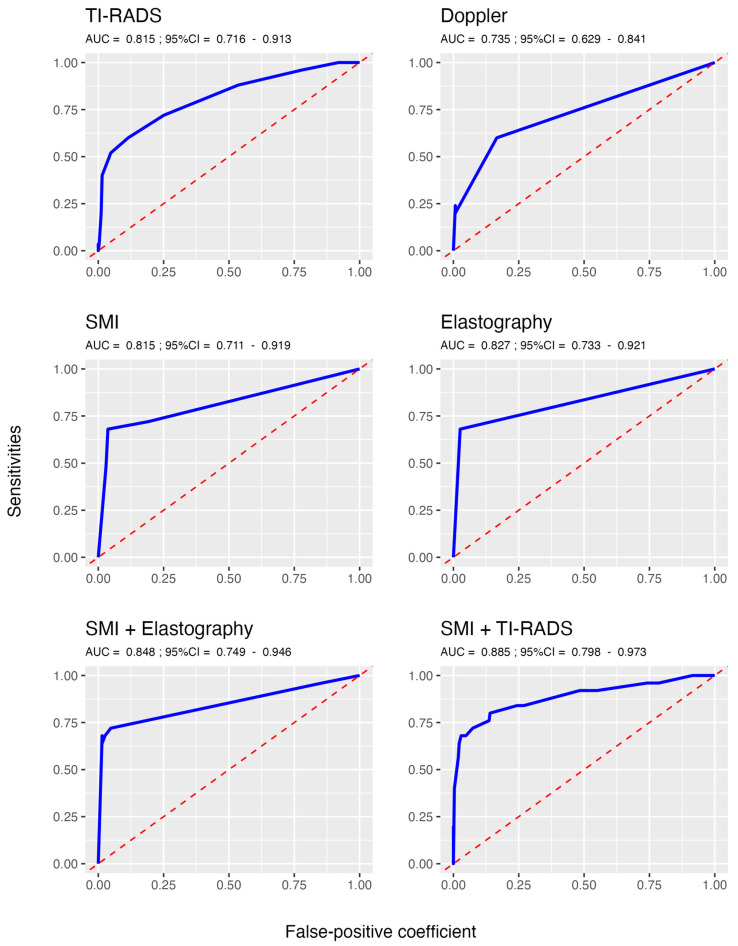
Receiver operating characteristic (ROC) curves for the continuous markers. TI-RADS: Thyroid Imaging Reporting and Data System; SMI: superb microvascular imaging.

Table 3 displays the scores derived from the corresponding logistic regression models, with cutoff values selected to maximize the Youden index. Figure 1 illustrates the receiver operating characteristic (ROC) curves for the continuous markers.

Table 4 presents the diagnostic performance parameters for each thyroid cancer diagnostic marker. Based on the Youden index, which is independent of sample size and economic cost considerations, the Doppler-only score exhibited the poorest performance, while the combinations of SMI with elastography and SMI with TI-RADS demonstrated the best performances, with comparable results.

Table 5 shows the gold-standard ($D_{1}$) and the thyroid cancer diagnostic rule based on elastography + SMI ($D_{2}$), its costs, and the Youden index. The corresponding utility functions are follows:

$$U_{1}\left(Gold-Standard\right)=\alpha +0.111\cdot \left(1-\alpha \right)$$

$$U_{2}\left(Elastography+SMI\right)=0.69\cdot \alpha +0.889\cdot \left(1-\alpha \right)$$

## 4. Discussion

This study’s main finding was that, among patients referred to the radiology department of a tertiary hospital in Spain for an evaluation of suspected cancerous thyroid lesions, the combination of elastography and superb microvascular imaging (SMI) achieved the highest diagnostic performance for thyroid cancer, with a Youden index of 0.69, a negative predictive value of 97.4%, and a positive predictive value of 69.1%. Additionally, we developed a novel utility function for this diagnostic rule, comparing it with the gold standard (FNA biopsy with cytopathologic diagnosis) based on the balance between diagnostic reliability and cost. This utility function allows clinicians to choose a reliable prognostic rule for thyroid cancer diagnosis, tailored to their prioritization of reliability versus cost-effectiveness.

This study included 300 patients, of whom 8.3% were newly diagnosed with thyroid cancer via biopsy and cytopathologic analysis, with only 4 (1.3%) having an uncertain diagnosis. After excluding these 4, 296 patients were analyzed, with 25 newly diagnosed with thyroid cancer, predominantly women, consistent with prior literature [2]. Although Europe reports a relatively high age-standardized thyroid cancer incidence (7.5 per 100,000) but low mortality (0.21 per 100,000) [34], the incidence in our cohort was lower.

To assess diagnostic performance and derive prognostic scores, all patients underwent conventional B-mode ultrasound [2,3] followed by elastography [24,26,27], color Doppler [7], SMI vascularization analysis [8,28], TI-RADS scoring [6,12,13,14,15], and FNA biopsy with Bethesda System for Reporting Thyroid Cytopathology (TBSRTC) assessment [16,17,29], culminating in a final pathologic report. Table 1 summarizes the patient characteristics for the entire cohort (*n* = 296) and subgroups with (*n* = 25) and without thyroid cancer. No statistically significant differences were found in age or thyroiditis diagnosis between subgroups, but men were significantly more likely to have thyroid cancer. Other comparisons between subgroups were also significantly different, as shown in Table 1, providing critical data for developing diagnostic rules.

Logistic regression models using Lasso-selected variables, with 95% confidence intervals and *p*-values derived from 1000 bootstrap replicates, identified intranodular vascularization as a cancer risk factor in both Doppler-only and SMI-only models; conversely, peripheral vascularization showed a protective effect in the “Doppler peripheral” and “SMI both” models. Intranodular vascularization, assessed by color Doppler, is a well-established ultrasound feature linked to higher malignancy risk, particularly in solid nodules with central hypervascularization, as noted in recent guidelines [13,35]. However, since 20–30% of benign nodules may also exhibit hypervascularization, guidelines recommend integrating it with other ultrasound features, such as elastography and SMI, as implemented in this study [36]. When all variables—TI-RADS, Doppler, SMI, and elastography—were included in the logistic model, Lasso retained elastography and SMI-detected intranodular vascularization. When elastography was excluded, the model incorporated TI-RADS with SMI-detected intranodular vascularization, highlighting elastography’s critical role in thyroid cancer diagnosis in our study.

The logistic regression model scores, with cutoff values optimized to maximize the Youden index for diagnostic rule performance, are presented in Table 3. A patient was classified as positive for thyroid cancer if their score exceeded the specified cutoff value shown in the table. Figure 1 illustrates the area under the ROC curves (AUC) for continuous markers, with the highest sensitivities and lowest false positives achieved by SMI combined with elastography (AUC = 0.84) and SMI combined with TI-RADS (AUC = 0.88).

The diagnostic performance parameters for each marker are summarized in Table 4. The Doppler-only score showed poor performance based on the Youden index, which evaluated diagnostic rule performance independently of sample size and cost. By contrast, SMI combined with elastography and SMI combined with TI-RADS were the top-performing rules (Youden index = 0.69), with comparable confidence intervals. While both predictors exhibited high negative predictive values (~97%), SMI plus elastography demonstrated a higher positive predictive value (69.1%) compared with SMI plus TI-RADS (47.5%).

The comparison between the gold standard (D1: FNA biopsy with cytopathologic diagnosis) and the elastography plus SMI rule (D2), based on real and normalized costs and the Youden index, is presented in Table 5. According to cost data from our hospital’s analytical accounting department, D1 is approximately eight times more expensive than D2. Figure 1 displays the utility functions for both diagnostic rules, using the weighting parameter alpha (α) to balance diagnostic reliability and cost effectiveness. The optimal decision rule maximized this utility function. D1 outperformed D2 when α exceeded 0.716, where their diagnostic values were equivalent. For α values near 1 (prioritizing reliability), D1 was preferred; for α values near 0 (emphasizing cost effectiveness), D2 was the optimal choice.

The main finding of this study is that, in patients who were referred to a radiology department of a tertiary hospital with suspicious cancerous thyroid lesions for assessment and who underwent FNA biopsies under US control followed by cytopathologic diagnosis and other additional thyroid US techniques, elastography plus SMI was the best diagnostic performance rule for the diagnosis of thyroid cancer based on a Youden index, which measured the performance of the diagnostic rule, of 0.69, with a high negative predictive value of 97.4 and a good positive predictive value of 69.1. In addition, we described a novel utility function for this diagnostic rule and compared it with the gold standard, FNA biopsies followed by cytopathologic diagnosis, based on the trade-off between diagnostic reliability and costs. This utility function will allow clinicians to choose a trustworthy prognostic rule for thyroid cancer diagnosis based on the weight given to reliability and cost.

We consider elastography combined with superb microvascular imaging (SMI) to be a complementary tool rather than a replacement for the gold standard (FNA biopsy with cytopathologic diagnosis), particularly for patients at high risk of thyroid cancer. A false negative result could have serious consequences, including delayed treatment and increased follow-up costs.

The diagnostic rule combining elastography with SMI demonstrated a high negative predictive value (NPV) of 97.4% (95% CI 95.7–98.9%). This NPV depends on the prevalence of thyroid cancer in the population studied. However, for an individual patient, a clinician can form a subjective estimate of the probability of thyroid cancer. This perceived likelihood could guide the decision to perform a FNA biopsy with cytopathologic diagnosis. By integrating the sensitivity and specificity of the proposed diagnostic test with the clinician’s subjective probability, Bayes’ theorem can be applied to calculate a posterior probability of cancer, which can refine the clinician’s initial assessment.

**Figure 2:**
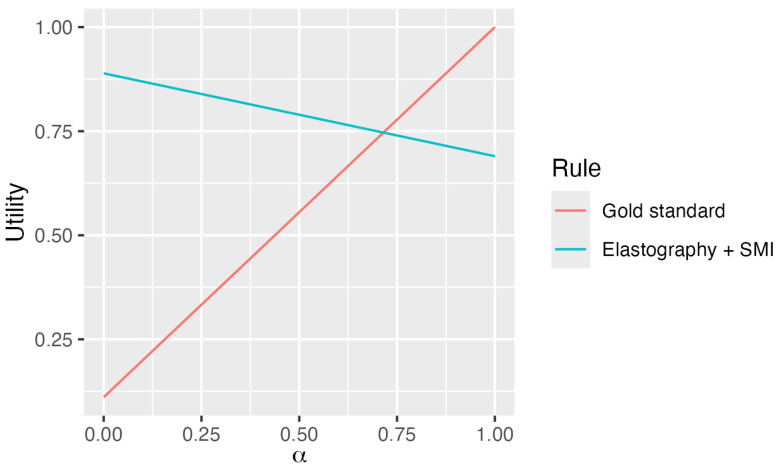
Utility functions of the thyroid cancer diagnostic rules based on the weighting parameter α Note that the gold standard outperforms the combination of elastography plus SMI for values of α greater than 0.716.

The cost–benefit utility function used is a compromise between the diagnostic safety of the rule evaluated through the Youden index and the economic cost. The parameter α (0 < α < 1) is the importance that each clinician attributes to the safety of the rule. The clinician can assign a value to the safety of each rule, for example, 90%. In this case, as can be seen in Figure 2, he/she should opt for the FNA biopsy with cytopathologic diagnosis rule, since its usefulness is greater than the elastography plus SMI rule.

This study’s strengths include its prospective, single-center design at a tertiary Spanish university hospital, ensuring systematic data collection and minimizing retrospective bias while enhancing internal validity through consistent procedures. Advanced ultrasound techniques (SWE, SMI, color Doppler, TI-RADS, and B-mode US) achieve high diagnostic performance (Youden index: 0.69; NPV: 97.4%). Comprehensive data on nodule characteristics (echogenicity, vascularity, and elastography) and standardized systems (TI-RADS and Bethesda) support robust diagnostic models. Rigorous patient selection, excluding inconclusive FNA results (Bethesda I and III), ensures reliable diagnoses. Lasso-penalized logistic regression and bootstrap analysis (1000 replicates) manage multicollinearity and high-dimensional data, strengthening predictive models. A novel utility function balancing diagnostic reliability and cost provides clinicians with a practical decision-making tool. These findings address the need for accurate, cost-effective thyroid cancer diagnostics, potentially reducing unnecessary procedures and optimizing clinical resources.

This study’s limitations include the low prevalence of thyroid cancer, which may limit the generalizability of the findings to populations with higher cancer incidence. All ultrasound assessments were conducted by a single expert radiologist, potentially introducing observer bias and reducing reproducibility in settings with less experienced radiologists. The study’s regional context, with low thyroid cancer prevalence, may not apply to areas with higher incidence or limited access to advanced imaging (e.g., SMI or Aplio™ 500 systems (Toshiba Medical Systems Corporation, Tochigi, Japan)). The small number of cancer cases (*n* = 25) restricts the robustness of subgroup analyses, particularly for variables such as sex or specific nodule characteristics. Finally, the diagnostic models require external validation beyond the study setting to confirm their applicability. We are currently collecting data in our hospital setting on thyroid cancer to validate the proposed diagnostic guidelines and enhance the generalizability of our findings.

## 5. Conclusions

We conclude that, in patients with suspected thyroid cancer lesions undergoing ultrasound-guided fine needle aspiration (FNA) biopsies followed by cytopathologic diagnosis, the combination of elastography and superb microvascular imaging achieved the strongest diagnostic performance for thyroid cancer. Additionally, we developed a utility function for this diagnostic rule, comparing it with the gold standard (FNA biopsy with cytopathologic diagnosis). This utility function enables clinicians to select a reliable diagnostic rule for thyroid cancer, tailored to their prioritization of reliability versus cost effectiveness.

## Figures and Tables

**Table 1:** Patient characteristics based on the presence or absence of a diagnosis of thyroid cancer by pathology diagnosis after FNA or Tru-cut biopsies.

		Thyroid Cancer		
	Overall N = 296	No N = 271	Yes N = 25	*p*-Value
Age (years)	58.4 ± 14.2	58.1 ± 14.3	61.7 ± 13.7	0.23
*Sex male*	74 (25.0)	63 (23.2)	11 (44.0)	0.022
Thyroiditis	47 (15.9)	44 (16.2)	3 (12.0)	0.777
Goiter	292 (98.7)	269 (99.3)	23 (92.0)	0.037
Carcinoma/lymphoma	24 (8.1)	7 (2.6)	17 (68.0)	<0.001
Papillary cancer	11 (3.7)	3 (1.1)	8 (32.0)	<0.001
Follicular cancer	8 (2.7)	3 (1.1)	5 (20.0)	<0.001
TI-RADS	4 (3; 5)	4 (3; 4.5)	7 (4; 8)	<0.001
TI-RADS > 4	86 (29.1)	68 (25.1)	18 (72.0)	<0.001
Thyroidectomy	24 (8.1)	14 (5.2)	10 (40.0)	<0.001
Elastography				<0.001
Benign	272 (91.9)	264 (97.4)	8 (32.0)	
Malignant	24 (8.1)	7 (2.6)	17 (68.0)	
*Doppler*				<0.001
Peripheral	240 (81.1)	226 (83.4)	14 (56.0)	
Intranodular	7 (2.4)	2 (0.7)	5 (20.0)	
Both	49 (16.6)	43 (15.9)	6 (24.0)	
*SMI*				<0.001
Peripheral	232 (78.4)	221 (81.5)	11 (44.0)	
Intranodular	21 (7.1)	8 (3.0)	13 (52.0)	
Both	43 (14.5)	42 (15.5)	1 (4.0)	
*NT composition*				0.011
Cystic/spongiform	63 (21.4)	62 (22.9)	1 (4.2)	
Mixed	58 (19.7)	56 (20.7)	2 (8.3)	
Solid	174 (59.0)	153 (56.5)	21 (87.5)	
*Bethesda System category*				<0.001
II, III	277 (94.5)	271 (100.0)	6 (27.3)	
IV, V, VI	16 (5.5)	0	16 (72.7)	
*TN echogenicity*				<0.001
Anechoic	17 (5.8)	17 (6.3)	0	
Isoechoic/hyperechoic	157 (53.2)	152 (56.1)	5 (20.8)	
Hypoechoic	109 (36.9)	93 (34.3)	16 (66.7)	
Very hypoechoic	12 (4.1)	9 (3.3)	3 (12.5)	
*TN. Shape*				<0.001
Smooth/well defined	258 (87.5)	243 (89.7)	15 (62.5)	
Loculated/irregular	17 (5.8)	10 (3.7)	7 (29.2)	
Extra-thyroid extension	20 (6.8)	18 (6.6)	2 (8.3)	
*TN. Echogenic foci*				0.041
None/artifacts	187 (63.4)	177 (65.3)	10 (41.7)	
Macrocalcifications	37 (12.5)	34 (12.5)	3 (12.5)	
Peripheral calcifications	37 (12.5)	32 (11.8)	5 (20.8)	
Punctate foci	34 (11.5)	28 (10.3)	6 (25.0)	
*Tru-cut biopsy*				<0.001
Yes	9 (3.0)	4 (1.5)	5 (20.0)	
No	287 (97.0)	267 (98.5)	20 (80.0)	

Data are means ± SDs, frequencies (%), and medians (IQRs). FNA: fine-needle aspiration; TI-RADS: Thyroid Imaging Reporting and Data System; SMI: superb microvascular imaging; TN: thyroid nodules; Peripheral Doppler: peripheral node vascularization identified by Doppler; Intranodular Doppler: internal node vascularization identified by Doppler; SMI peripheral: peripheral vasculature identified by SMI; SMI Intranodular: internal node vasculature identified by SMI; SMI. Both: SMI-detected intranodular and peripheral vascularization.

**Table 2:** Multivariate logistic regression for thyroid cancer using the Lasso-selected variables.

Mechanism	Selected Variables	Coefficient (95% CI) *	*p*-Value *
Doppler only	Doppler. Peripheral	−2.399 (−4.153; −0.227)	<0.001
	Doppler. Intranodular	0.826 (0.000; 1.697)	0.001
SMI only	SMI. Intranodular	3.614 (0.562; 4.764)	<0.001
	SMI. Both	−3.491 (−5.042; −0.657)	<0.001
SMI + elastography (if all 4 variables are included)	Malignant elastography	3.611 (1.832; 5.364)	<0.001
	SMI. Intranodular	0.735 (0.000; 2.769)	0.03
	SMI. Both	−1.395 (−3.349; 0.000)	0.009
SMI + TI-RADS (if only elastography is excluded)	TI-RADS	0.402 (0.167; 0.708)	<0.001
	SMI. Intranodular	3.020 (0.728; 4.585)	0.001
	SMI. Both	−3.170 (−5.390; −0.462)	<0.001

(*) The confidence intervals and *p*-values were derived using a bootstrap resampling procedure (1000 replicates); Lasso: Least Absolute Shrinkage and Selection Operator; Peripheral Doppler: peripheral vascularization identified by Doppler; Intranodular Doppler: internal node vascularization identified by Doppler; SMI: superb microvascular imaging; SMI. Both: SMI-detected intranodular and peripheral vascularization. TI-RADS: Thyroid Imaging Reporting and Data System.

**Table 3:** Prognostic scores for thyroid cancer.

Mechanism	Score	Cutoff *
TI-RADS	-	5.5
Doppler only	−2.399×Doppler.Peripheral+0.826×Doppler.Intranodular	−1.986
SMI only	3.614×SMI.Intranodular−3.491×SMI.Both	1.845
SMI + elastography	3.611×Elastography+0.735×SMI.Intranodular−1.395×SMI.Both	0.367
SMI + TI-RADS	0.402×TI−RADS+3.020×SMI.Intranodular−3.170×SMI.Both	2.61

(*) A patient can be considered positive for thyroid cancer if and only if the score is above the corresponding cutoff point. TI-RADS: Thyroid Imaging Reporting and Data System; SMI: superb microvascular imaging.

**Table 4:** Diagnostic performance parameters for the diagnosis of thyroid cancer.

	TI-RADS	Doppler Only	SMI only	Elastography	SMI + Elastography	SMI + TI-RADS
Youden index *	0.52 (0.34; 0.70)	0.43 (0.24; 0.62)	0.64 (0.44; 0.83)	0.65 (0.48; 0.83)	0.69 (0.50; 0.85)	0.69 (0.52; 0.84)
Accuracy	86.8 (69.9; 94.3)	81.4 (77.0; 86.5)	93.9 (90.5; 96.6)	94.9 (91.6; 97.0)	94.9 (91.2; 97.6)	90.9 (77.7; 96.6)
Sensitivity	64.0 (44.0; 88.0)	60.0 (40.0; 80.0)	68.0 (48.0; 88.0)	68.0 (46.4; 84.3)	72.0 (52.0; 88.0)	76.0 (60.0; 92.0)
Specificity	88.9 (69.4; 97.80)	83.4 (78.6; 88.2)	96.3 (93.7; 98.5)	97.4 (94.5; 98.9)	97.0 (93.4; 99.6)	92.3 (76.8; 98.9)
PPV	34.9 (17.3; 70.8)	25.0 (17.7; 35.3)	63.3 (45.4; 80.8)	70.8 (48.8; 86.6)	69.1 (48.5; 94.7)	47.5 (25.6; 84.20)
NPV	96.5 (94.7; 98.6)	95.8 (93.8; 97.8)	97.0 (95.3; 98.8)	97.1 (94.1; 98.6)	97.4 (95.7; 98.9)	97.8 (96.2; 99.3)

(*) **The diagnostic rule was chosen based on the Youden index and economic cost considerations.** TI-RADS: Thyroid Imaging Reporting and Data System; SMI: superb microvascular imaging; PPV: positive predictive value; NPV: negative predictive value.

**Table 5:** Thyroid cancer diagnostic rules, costs, and the Youden index.

	Costs		Youden
Rule diagnosis	Real (EUR)	Normalized	Estimated
$D_{1}=$ Gold Standard: FNA-Tru-cut Biopsies	302.44	0.889	1
$D_{2}$ = Elastography + SMI	37.74	0.111	0.69

FNA: fine-needle aspiration; SMI: superb microvascular imaging.

## Data Availability

Please contact the authors for data requests.

## Abbreviations

The following abbreviations are used in this manuscript:

US	ultrasonography
FNAB	fine-needle aspiration biopsy
TI-RADS	Thyroid Imaging Reporting and Data System
SMI	superb microvascular imaging
SWE	shear wave elastography
TBSRTC	Bethesda System for Reporting Thyroid Cytopathology
